# Improving the identification of bone‐specific physical activity using wrist‐worn accelerometry: A cross‐sectional study in 11–12‐year‐old Australian children

**DOI:** 10.1002/ejsc.12122

**Published:** 2024-05-04

**Authors:** Gemma Brailey, Brad Metcalf, Lisa Price, Sean Cumming, Alex Rowlands, Timothy Olds, Peter Simm, Melissa Wake, Victoria Stiles

**Affiliations:** ^1^ Department of Public Health and Sport Sciences Faculty of Health and Life Sciences University of Exeter Exeter UK; ^2^ Department for Health University of Bath Bath UK; ^3^ Diabetes Research Centre Leicester General Hospital University of Leicester Leicester UK; ^4^ National Institute for Health Research (NIHR) Leicester Biomedical Research Centre (BRC) University Hospitals of Leicester NHS Trust and the University of Leicester Leicester UK; ^5^ Alliance for Research in Exercise, Nutrition, and Activity University of South Australia Adelaide South Australia Australia; ^6^ Murdoch Children's Research Institute Royal Children's Hospital Parkville Victoria Australia; ^7^ Department of Endocrinology and Diabetes Royal Children's Hospital Parkville Victoria Australia; ^8^ Department of Paediatrics University of Melbourne Parkville Victoria Australia

**Keywords:** accelerometry, adolescents, bone health, cross‐sectional studies, physical activity

## Abstract

Physical activity (PA) during childhood and adolescence is important for the accrual of maximal peak bone mass. The precise dose that benefits bone remains unclear as methods commonly used to analyze PA data are unsuitable for measuring bone‐relevant PA. Using improved accelerometry methods, this study identified the amount and intensity of PA most strongly associated with bone outcomes in 11–12‐year‐olds. Participants (*n* = 770; 382 boys) underwent tibial peripheral quantitative computed tomography to assess trabecular and cortical density, endosteal and periosteal circumference and polar stress‐strain index. Seven‐day wrist‐worn raw acceleration data averaged over 1‐s epochs was used to estimate time accumulated above incremental PA intensities (50 milli‐gravitational unit (m*g*) increments from 200 to 3000 m*g*). Associations between time spent above each 50 m*g* increment and bone outcomes were assessed using multiple linear regression, adjusted for age, sex, height, weight, maturity, socioeconomic position, muscle cross‐sectional area and PA below the intensity of interest. There was a gradual increase in mean *R* ^2^ change across all bone‐related outcomes as the intensity increased in 50 m*g* increments from >200 to >700 m*g*. All outcomes became significant at >700 m*g* (*R* ^2^ change = 0.6%–1.3% and *p* = 0.001–0.02). Any further increases in intensity led to a reduction in mean *R* ^2^ change and associations became non‐significant for all outcomes >1500 m*g*. Using more appropriate accelerometry methods (1‐s epochs; no a priori application of traditional cut‐points) enabled us to identify that ∼10 min/day of PA >700 m*g* (equivalent to running ∼10 km/h) was positively associated with pQCT‐derived measures of bone density, geometry and strength in 11–12‐year‐olds.

## Highlights

- Accelerometry methods that are commonly used to assess free‐living, bone‐relevant physical activity (PA) are not well suited to measuring bone‐specific characteristics of PA (short bursts of high‐impact activity).
- Processing raw acceleration data using much shorter epochs (1‐s) and using incremental 50 m*g* intensity thresholds (rather than traditional, broad intensity thresholds) enabled a bone‐specific, beneficial intensity to be identified.
- The methods outlined provide a means for more precisely assessing free‐living bone‐relevant PA and permit the transparent and reproducible analysis of raw accelerometry data, which will enhance our ability to quantify dose‐response associations in the future.

## 1 INTRODUCTION

Globally, osteoporosis is responsible for 8.9 million fractures per annum and conveys substantial social and economic burden (Johnell et al., 2006). A large proportion of osteoporosis risk (∼60%) is determined by the amount of bone accrued at the point of peak bone mass (PBM) in early adulthood (Bonjour et al., 2009). Whilst non‐modifiable genetic factors account for a large degree of the variability in PBM (60%–80%), one of the most important modifiable influences is physical activity (PA) experienced during childhood and adolescence (Weaver et al., 2016). However, identification of specific doses of PA that confer benefits to bone health remains problematic (Weaver et al., 2016).

Accelerometers have become the method of choice for assessing PA in relation to health outcomes (Trost, 2020) with the wrist wear‐location becoming increasingly popular due to the improved wear compliance and more complete depictions of free‐living PA that are obtained (Fairclough et al., 2016). However, the accelerometry methods most widely used in studies assessing PA‐bone relationships average proprietary outputs over long (e.g., 15–60‐s) epochs and classify this output into intensity categories (e.g., moderate PA [MPA] and vigorous PA [VPA]), using cut‐points (numerous) that are selected at the outset (Brailey et al., 2022). As a result, the short bursts of high‐intensity activity that are required to initiate osteogenesis (Turner et al., 2003) and sporadically performed by children (Bailey et al., 1995; Baquet et al., 2007) are “over‐smoothed” and misclassified as lower intensity activity (Nilsson et al., 2002). Further, different cut‐points result in divergent PA estimates (Freedson et al., 2005; Routen et al., 2012) and prevent direct comparison between studies (Bornstein et al., 2011). Thus, a crucial opportunity to aggregate data and identify important trends between PA, health and disease is missed (Bornstein et al., 2011). Although familiar to researchers, the continued use of these methods contributes to the lack of clarity regarding the dose‐response relationship between PA and bone health in youth.

To improve the identification of bone‐specific PA in free‐living situations, a recent systematic review of studies assessing accelerometer‐derived habitual PA in relation to bone outcomes recommended that shorter 1‐s epochs be used to ensure that brief dynamic episodes of osteogenic activity are captured more in their entirety (Brailey et al., 2022). It was also stated that a data‐driven approach that identifies the intensities of activity most strongly associated with bone outcomes may be more informative than investigating the associations with pre‐defined intensity cut‐points (such as MPA, moderate‐vigorous PA (MVPA), and VPA) that are calibrated against measures of energy expenditure (Brailey et al., 2022). An example of this approach already exists in a study of adult women from UK Biobank (Stiles et al., 2017). Open‐source software was used to process raw acceleration from a wrist‐worn device in 1‐s epochs and instead of applying cut‐points a priori, time accumulated in incremental intensities of acceleration (50 milli‐gravitational units (m*g*)) was used to identify that on average, only a few minutes of activity/day above 750 and 1000 m*g* (found to be equivalent to slow‐medium paced running) was associated with bone health in post‐ and pre‐menopausal women, respectively. Using this approach, an estimate of the volume of PA accumulated can also be obtained (Stiles et al., 2018). This technique gives more value to brief periods of high‐intensity activity in the analysis, a characteristic of PA, which may be particularly pertinent to generating an osteogenic response (Stiles et al., 2018), especially in children where PA tends to be accumulated in short bursts (Bailey et al., 1995; Baquet et al., 2007).

As the opportunity to accrue bone is enhanced during growth, the amount and intensity of habitual PA found to benefit bone is likely to differ in children and adolescents compared to adults (Bailey et al., 1999; Baxter‐Jones et al., 2011) and warrants further investigation. Large scale observational studies, such as Australia's Child Health CheckPoint (Clifford et al., 2019), have been instrumental in demonstrating that significant associations between bone outcomes and habitual PA measured using a wrist‐worn accelerometer in children do exist, with higher activity levels resulting in greater bone mass, geometry and strength (Osborn et al., 2018), contributing to a reduction in fracture risk (Kalkwarf et al., 2011). However, the use of traditional intensity classifications (e.g., MVPA) and long epoch lengths has prevented the precise amount and intensity of PA required to benefit bone health from being identified (Brailey et al., 2022). Therefore, this study aims to use open‐source software to process and analyze raw acceleration data using methods that have been modified to better detect bone‐relevant PA to identify the intensity of acceleration most strongly associated with pQCT‐derived measures of bone health in a large cross‐sectional study of 11–12‐year‐olds.

## 2 MATERIALS AND METHODS

### 2.1 Study population

Participants were part of the Child Health CheckPoint (Clifford et al., 2019), a cross‐sectional physical health and biomarker module, that was embedded within the population‐based Longitudinal Study of Australian Children (LSAC) (Edwards, 2014). The LSAC and CheckPoint studies are described in detail elsewhere (Clifford et al., 2019; Edwards, 2014). In 2004, a two‐stage cluster randomized design was used to recruit a nationally representative cohort of 5107 infants (B cohort) to LSAC. Children and families enroled in LSAC are followed biennially with 6 data collection waves completed up until 2015 (73.7% retention rate). CheckPoint data collection took place from February 2015 to March 2016 between LSAC's sixth and seventh waves when B cohort children were 11–12‐years‐old. Written informed consent for LSAC was provided by the child's parent/guardian. At the beginning of wave 6, families provided written consent to be contacted by the CheckPoint team to undertake additional testing.

From December 2014, consenting families were invited to attend either a main assessment center, mini assessment center or home visit. Details of each assessment center and measures conducted are in the CheckPoint Data User Guide (Clifford et al., 2018). Since bone measures were only taken at the main assessment center, only participants who attended the main assessment center were eligible for inclusion in this study. Study data were collected and managed using Research Electronic Data Capture (REDCap) electronic data capture tools (Harris et al., 2009, 2019) hosted at the Murdoch Children's Research Institute.

### 2.2 Anthropometric measures

Anthropometric measures were conducted in line with previous methods (Marfell‐Jones et al., 2006). Standing height was measured (to the nearest 0.1 cm) using a portable stadiometer (Model IP0955, Invicta) with the head in the Frankfort plane and shoes and socks removed. Two measures were taken and if these differed by >0.5 cm, a third was conducted. The average of the two closest measurements was reported. Weight was measured once (to the nearest 0.1 kg) using four‐limb bioelectrical impendence analysis scales (InBody230 scales; InBody Co Ltd.). Children wore light clothing, removed shoes and socks and held the two horizontal handles whilst standing on the scales. Body mass index (kg/m^2^) was calculated using weight (kg)/height (m)^2^.

### 2.3 Pubertal assessment

Pubertal status was self‐reported using the Pubertal Development Scale (PDS; Petersen et al., 1988), and participants were assigned to pre‐, early, mid‐, late and post‐pubertal categories. The PDS is a validated, reliable method of assessing pubertal status (Brooksgunn et al., 1987; Petersen et al., 1988) and asks questions relating to growth spurt in height and changes in body hair and skin in both males and females, along with sex‐specific questions regarding deepening of voice and facial hair in males and breast growth and menarche in females.

### 2.4 Socioeconomic status

Socio‐economic status was assessed using The Australian Bureau of Statistics' Socio‐economic Indicators for Areas (SEIFA) 2011 Index of Relative Socio‐economic Disadvantage Score (Statistics ABo, 2011). This census‐based measure was determined from each participant's postcode and considers household education level, income, employment status and disability. A higher score indicates greater advantage. The populations mean score for Australia is 1000 with an standard deviation (SD) of 100.

### 2.5 Bone health assessment

One of the three licensed research assistants performed a peripheral quantitative computed tomography (pQCT) scan of the non‐dominant distal tibia (dominance classified as the leg preferentially used to kick a ball) using a single Stratec XCT 2000L pQCT scanner (Medizintechnik); methods and descriptive values have been reported elsewhere (Vlok et al., 2019). Participants were allocated 7 min for one scout scan and two research scans of the lower leg at the relative distances of 4% and 66%, respectively. Quality control was measured daily using a standard phantom (XCT2000L, 0.370 1/cm) and every 30 days using a cone phantom. A standard measuring tape was used to manually assess tibial length. This was defined as the distance between the superior edge of the medial malleolus and the medial edge of the tibial plateau (Moyer‐Mileur et al., 2008) and was identified through palpation and marked with a pen. Seated participants then extended their non‐dominant leg through the scanner gantry and their foot (shoeless) was secured to the pQCT footrest. As the scanner gantry was only 14 cm in diameter, participants with larger calves had scans at the 4% site only. A scout scan identified the distal epiphyseal plate and allowed placement of a reference line at its proximal edge. The 4% (ankle) and 66% (shin) sites of the tibia were then identified by the researcher using this reference line in relation to limb length. One tomographic image was taken at each site (scan speed 20 mm/s, slice thickness 2.4 mm, and voxel size 0.4 mm).

Using Stratec XCT 2000 software (version 6.20C), one of two trained research assistants reviewed the regions of interest around the total image at the 4% and 66% sites and adjusted where necessary. The MACRO analysis function was then used to generate bone measures with near perfect inter‐rater agreement (ICC >0.99). At the 4% distal cross‐section, trabecular bone mineral density (BMD; m*g*/cm^3^) was calculated using a threshold of 169.0 m*g*/cm^3^, peel and contour modes: 1 and a trabecular area of 45% (Biggin et al., 2013). At the 66% proximal cross‐section, cortical BMD (m*g*/cm^3^), endosteal circumference (mm) and periosteal circumference (mm) were calculated using a threshold of 710 g/cm^3^, contour mode: 1 and peel and cortical modes: 2 (Farr et al., 2011; Sherk et al., 2012). The polar stress‐strain index (SSI; mm^3^) was calculated at the 66% site from geometry and density measures (threshold = 480.0 m*g*/cm^3^, peel and contour modes = 1 and filter = F01 (Brookes et al., 2015)). Muscle cross‐sectional area (MCSA) was calculated by subtracting the CSA of bone from the combined muscle and bone CSA (threshold 50–540 m*g*/cm³, contour mode: 3, peel mode: 1 and filter: F03F05 (Brookes et al., 2015; Sherk et al., 2012)). Each scan was reviewed and using methods similar to Blew et al. (2014) given a quality score of 1 (best) to 5 (worst) based on image resolution, presence of motion artifacts and a clearly definable region of interest. Images were excluded if they were graded 4 or 5 and had motion artifact that affected the region of interest (inner 45% of trabecular bone in 4% scans or cortical bone in 66% scans).

### 2.6 PA monitoring and data processing

At the end of the visit, participants were given a wrist‐worn triaxial GENEActiv accelerometer (dynamic range ±8 *g*, where *g* = the value of gravity (9.81 m/s^2^); Activinsights Ltd.); methods and descriptive values have been reported elsewhere (Fraysse et al., 2019). Monitors were configured using the GENEActiv PC software (version 2.9) to capture and store raw acceleration at 50 Hz for 14 days. Participants were instructed to wear the accelerometer on the non‐dominant wrist continuously for 8 days (wear started on the day of the visit, but recording did not begin until midnight), removing only for prolonged water immersion (swimming and bath) or sports where they were not permitted. Devices were returned in a pre‐paid postal envelope.

Data were downloaded (GENEActiv PC software; version 2.9 Activinsights Ltd.) and saved in raw format as .bin files. The open‐source R‐package GGIR Version 1.9‐0 in R (http://cran.r‐project.org) (van Hees et al., 2013, 2014) was used to analyze accelerometer files. Processing of accelerometer data in GGIR involves autocalibration using local gravity as a reference (van Hees et al., 2014), detection of sustained abnormally high values, non‐wear detection and calculation of the average magnitude of dynamic acceleration corrected for gravity (Euclidean Norm minus 1 g; ENMO) (van Hees et al., 2013). ENMO values were averaged over 1‐s epochs and expressed in milli‐gravitational units (m*g*). To ensure any activity registered due to movement during travel/postage was not treated as meaningful activity, the GGIR script was restricted to 7 days to cover the main wear period. Accelerometer files that had a post‐calibration error >0.01 m*g* or less than 4 valid days wear (3 weekdays and 1 weekend day) were excluded. A valid day was defined as ≥16 h of wear during a 24‐h period (Rowlands et al., 2016). Non‐wear was identified when the SD of at least two of the three axes was <13 m*g*, and the value range was <50 m*g* assessed over 60 min windows with a 15‐min sliding window (da Silva et al., 2014). By default, when non‐wear was detected, invalid data were imputed by average data at similar time points from other days (described in detail elsewhere (van Hees et al., 2013)).

To avoid the application of any cut‐points to ENMO a priori, the time (minutes) accumulated in bins defined by incremental acceleration thresholds (10 m*g* increments from 0 to 50 m*g*; 50 m*g* increments from 50 to 3000 m*g*, for example, 50–99.99, 100–149.99, 150–199.99, … 3000 and in a single large bin from 3000 to 8000 m*g* (occurrence of time accumulated >3000 m*g* was rare)) was calculated for each day and averaged across all valid days. Time spent in each incremental acceleration bin for this “average day” was used to compile PA predictor variables of interest and respective covariates to assess associations with bone outcomes. Acceleration ranges representing a variety of activities were used to provide context and aid in the interpretation of activities that the most strongly associated intensity may represent (described previously in Rowlands et al. (2019)). Representative activities included walking (slow: ∼3 km/h, 100–200 m*g*; brisk: ∼5 km/h, >200–350 m*g* and fast: ∼6.5 km/h, >350–500 m*g*), running (slow: ∼8 km/h, >500–1000 m*g*; medium: ∼10 km/h, >1000–1500 m*g* and fast: ∼15 km/h, >1500–2000 m*g*) and sprinting/jumping (>2000 m*g*) (Rowlands et al., 2019). For comparison, published thresholds for MVPA and VPA for children range from 200–250 and 700–750 m*g*, respectively (Hildebrand et al., 2014; Phillips et al., 2013). To give value to short amounts of high‐intensity activity that are important for osteogenesis, a volume acceleration metric was also calculated from intensity and duration. The time in minutes in each 50 m*g* bin was multiplied by the average intensity of that bin (e.g., 725 m*g* was the average intensity for the 700–750 m*g* bin) to approximate the volume of each bin in m*g* minutes (Stiles et al., 2018). The bins were then summed to create a total volume variable for each threshold analyzed.

### 2.7 Statistical analyses

Descriptive statistics were calculated for anthropometric, bone and PA variables using mean (SD). Independent *t*‐tests were conducted to identify sex differences in anthropometric and bone variables. To identify the PA intensity most strongly and consistently associated with bone outcomes, the associations between each bone variable and the time spent above each incremental PA intensity threshold was examined using multiple linear regression. For each bone outcome, we ran 19 separate regression models each with a different PA intensity threshold. Each of these models was compared to the “covariate‐only” model, and the corresponding *R* ^2^ change and *p*‐values were recorded. The 19 intensity thresholds that we tested separately were >200, >250, >300, >350, >400, >450, >500, >550, >600, >650, >700, >750, >800, >850, >900, >950, >1000, >1500, and >3000 m*g* (designed to include accelerations that are typical of walking, through to sprinting and jumping in children (Rowlands et al., 2019)). This was done for each of the 5 bone outcomes to examine how the strength of associations (*R* ^2^ changes) varied between thresholds and to use these to identify the point at which the association between a threshold of acceleration was consistently the strongest across all 5 bone outcomes. The optimal intensity was determined by the magnitude of the mean *R* ^2^ change (calculated from the *R* ^2^ change for each bone outcome per intensity increment), the SD of the *R* ^2^ changes across all 5 bone outcomes per intensity increment, and on the number of bone outcomes that were significantly associated with each intensity increment.

Model 1 consisted of all potential covariates: age, sex, height, weight, pubertal status, SEIFA index and MCSA, which were selected a priori based on evidence of their influence on both PA and bone during growth (Cooper et al., 2015; Osborn et al., 2018; Weaver et al., 2016), and PA below the intensity of interest (e.g., 40–199 m*g* when intensity of interest >200 m*g*). Activity <40 m*g* was omitted to avoid multicollinearity and because PA <40 m*g* was considered non‐meaningful for bone outcomes (Osborn et al., 2018). When investigating higher intensities (e.g., >700 m*g*), PA covariates below the intensity of interest were split into smaller categories (e.g., activity classed as MPA (200–699 m*g* (Hildebrand et al., 2014; Phillips et al., 2013)) was divided into 200–399 m*g* and 400–699 m*g*) to account for activity at the lower or higher end of this category that may differ in impact magnitude and osteogenic potential. Model 2 included all variables entered in Model 1 with the addition of the intensity threshold of interest (e.g., >200 m*g* or >250 m*g* etc.). As there were no significant interactions of PA with maturity or sex, model coefficients are presented for boys and girls and all maturity groups combined. Residual plots were inspected for normality, linearity and homoscedasticity, and the variance inflation factor was used to assess whether there was any collinearity with the variable of interest. We did not adjust for multiple testing as we were not testing multiple PA variables, we were testing the same PA variable in each model just with different thresholds. The purpose was not to identify which PA variables were statistically significant and which were not but to record the magnitude of association (*R* ^2^ change) for each threshold and visually inspect a plot to identify the “optimal” threshold (akin to Receiver Operating Characteristic curve analyses where the true positive/false positive rate is recorded and plotted for each threshold on the classification scale in order). All analyses were conducted in IBM SPSS Version 27 (IBM).

## 3 RESULTS

### 3.1 Participant characteristics

**FIGURE 1:**
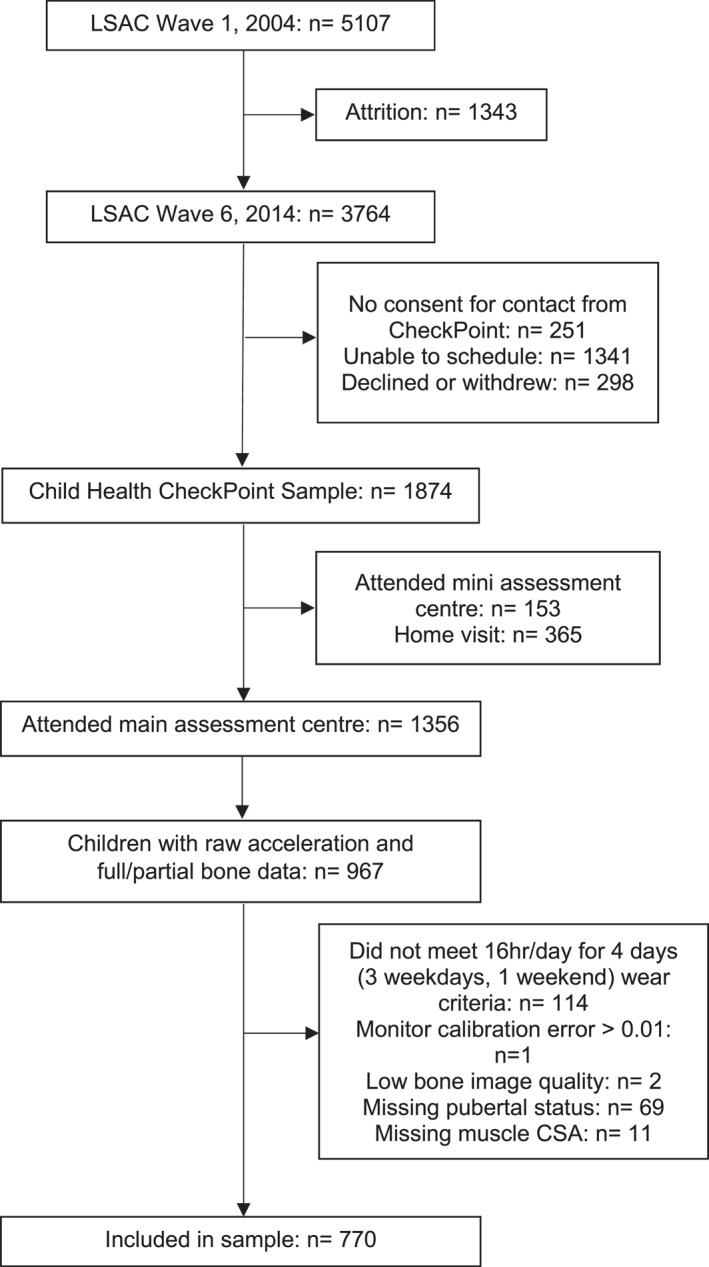
Participant flow chart. CSA, cross‐sectional area; LSAC, Longitudinal Study of Australian Children (Gray et al., 2005).

Of the 3764 children that participated in wave 6 of LSAC, 1874 attended the Child Health CheckPoint. Of those attending main assessment centers (*n* = 1356), 967 children had full or partial pQCT data (93% full and 7% partial) and raw acceleration files. A further 197 participants were excluded due to lack of covariate data (PDS and MCSA), poor bone image quality (graded 4 or 5), failure to meet the accelerometer wear criteria of 16 h/day for 4 days (3 weekdays and 1 weekend day) or due to acceleration files having a post‐calibration error >0.01 *g*. A total of 770 children (382 boys) were included in the final sample. Participant flow and reasons for exclusion are detailed in Figure 1. Participants in the present study had similar descriptive characteristics to those in the Child Health CheckPoint who did not satisfy the study inclusion criteria (*n* = 918; 484 boys). Girls in the present study were significantly taller than boys (*p* = 0.04) and had significantly higher cortical and trabecular BMD (*p* < 0.001 and *p* = 0.008) but significantly lower endosteal and periosteal circumferences (*p* < 0.001 and *p* = 0.006). Girls were also at a more advanced pubertal stage than boys with a lower proportion categorized as pre‐pubertal (5.4% vs. 16.5%) and higher proportion categorized as late/post pubertal (21.4% vs. 3.7%). Descriptive characteristics are presented in Table 1.

**TABLE 1:** Descriptive characteristics and peripheral quantitative computed tomography bone outcomes.

	All (*n* = 770)	Boys (*n* = 382)	Girls (*n* = 388)
Age (years)	11.9 (0.4)	11.9 (0.4)	11.9 (0.4)
Body height (cm)	153.1 (7.9)	152.5 (8.0)^a^	153.7 (7.9)
Body mass (kg)	44.8 (10.3)	44.2 (10.6)	45.4 (10.0)
BMI (kg/m^2^)	19.0 (3.4)	18.9 (3.5)	19.1 (3.2)
Puberty *N* [%]			
Pre‐pubertal	84 [10.9]	63 [16.5]	21 [5.4]
Early/mid‐pubertal	589 [76.5]	305 [79.9]	284 [73.2]
Late/post‐pubertal	97 [12.6]	14 [3.7]	83 [21.4]
Disadvantage (SEIFA)	1028 (60)	1030 (60)	1027 (61)
Bone and muscle measurements from pQCT			
Cortical BMD (m*g*/cm³)^b^	1018.9 (37.4)	1010.5 (35.7)^a^	1027.1 (37.2)
Trabecular BMD (m*g*/cm³)^c^	197.7 (25.9)	195.2 (23.4)^a^	200.2 (27.8)
Endosteal circumference (mm)^b^	61.9 (8.3)	63.0 (8.4)^a^	60.9 (8.1)
Periosteal circumference (mm)^b^	81.2 (7.2)	81.9 (7.5)^a^	80.5 (6.9)
Polar stress‐strain index (mm³)^b^	1700 (387)	1709 (396)	1692 (378)
Muscle CSA (mm^2^)^b^	4362 (704)	4313 (725)	4410 (682)

*Note*: Values are presented as mean (SD) unless otherwise indicated.

Abbreviations: BMD, volumetric bone mineral density; BMI, body mass index; pQCT, peripheral quantitative computed tomography; SEIFA, Socio‐Economic Indexes for Areas (national average = 1000 and SD = 100).

^a^ A significant difference between boys and girls, *p* < 0.05.

^b^ *n* = 770.

^c^ *n* = 762.

### 3.2 Associations between bone outcomes and time spent above incremental intensity thresholds

**FIGURE 2:**
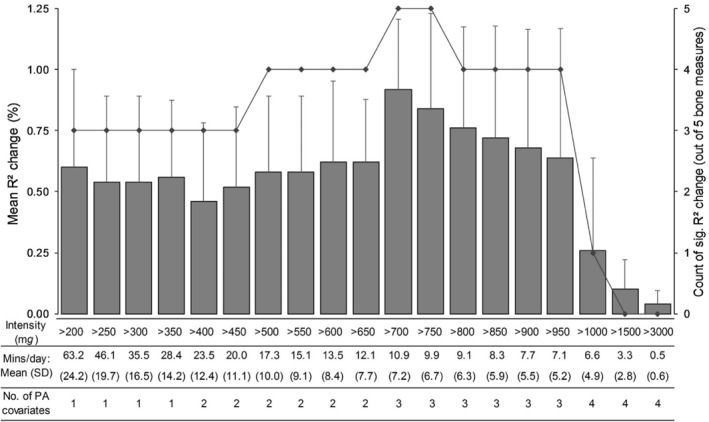
Mean *R* ^2^ change (%) (bar chart, error bars = standard deviation [SD]) for model 2 for all 5 bone outcomes (cortical density, trabecular density, endosteal circumference, periosteal circumference and polar stress‐strain index) from regression analyses with each 50 m*g* physical activity (PA) intensity increment from >200 to >1000 m*g* and for >1500 and >3000 m*g* intensities (model 1 adjusted for age, sex, height, weight, pubertal status, Socio‐economic Indicators for Areas index, muscle cross‐sectional area and PA intensities below the threshold of interest (from 40 m*g* onwards)). Model 2 = model 1 + PA intensity of interest. The line graph demonstrates the number of bone outcomes (out of 5) that were significantly associated with each intensity. The mean (SD) minutes/day for the whole sample as well as the number of PA covariates used in analyses for each intensity are presented below the chart. PA covariates used: 1 = 40 m*g*—intensity of interest; 2 = 40–199 m*g* + 200 m*g*—intensity of interest; 3 = 40–199 m*g*, 200–399 m*g* + 400 m*g*—intensity of interest and 4 = 40–199 m*g*, 200–399 m*g*, 400–699 m*g* + 700 m*g*—intensity of interest.

All assumptions for the regression analyses were met. The mean *R* ^2^ change across all 5 bone outcomes, the number of outcomes that were significant from the regression analyses and the respective time spent above each 50 m*g* intensity increment are displayed in Figure 2. As the PA intensity threshold of interest increased in 50 m*g* increments from >200 to >700 m*g*, there was a gradual increase in mean *R* ^2^ change across the five bone outcomes. For example, when adding PA >200 m*g* or >500 m*g* or >700 m*g* to the covariate model, the mean *R* ^2^ change was 0.54%, 0.58% and 0.92% with 3/5, 4/5 and 5/5 of the bone outcomes being *p* < 0.05, respectively. Further incremental increases in PA intensity thresholds from this point resulted in a reduction in overall mean *R* ^2^ change and associations became non‐significant for all bone outcomes from >1500 m*g* (mean *R* ^2^ change = 0.10%, *p* = 0.1–0.9; Figure 2). The *R* ^2^ change, unstandardized beta coefficients and associated *p* values for each bone outcome and PA >700 m*g* are presented in Table 2. The >700 m*g* threshold was associated with higher (i.e., better) trabecular BMD, bigger endosteal and periosteal circumference (bone size) and higher polar SSI (torsional strength). However, a negative relationship was identified with cortical BMD (unstandardized beta = −1.3, *p* = 0.001; Table 2.). As a sensitivity analysis, the regression analyses were repeated without including any PA covariates below the threshold of interest and with all PA below the intensity of interest collapsed into one covariate. Results followed the same pattern with PA >700 m*g* still having a larger overall mean *R* ^2^ change compared to the other PA intensities examined. Due to some sports not permitting accelerometer wear, sensitivity analyses removing those reporting more than on average 20, 30, and 60 min/d in sport (*n* = 562, 500 and 328, respectively) were conducted. Results followed the same pattern as the whole sample findings with the >700 m*g* threshold achieving the highest overall mean *R* ^2^ change. The mean time spent in activity >700 m*g* was 10.87 min/day.

**TABLE 2:** *R* ^2^ change, unstandardized beta coefficients and *p* values from multiple linear regression analyses.

Bone outcome	>700 m*g*			700–1500 m*g*		
	∆*R* ^2^ (%)	*B*	*p*	∆*R* ^2^ (%)	*B*	*p*
Cortical density^a^	1.3	−1.3	0.001	1.4	−2.7	<0.001
Trabecular density^b^	1.1	0.8	0.001	0.8	1.4	0.006
Endosteal circumference^a^	0.6	0.2	0.020	1.0	0.5	0.002
Periosteal circumference^a^	0.9	0.2	0.001	1.2	0.5	<0.001
Polar SSI^a^	0.7	9.7	<0.001	0.8	20.0	<0.001
Mean ± SD *R* ^2^ change	0.92 ± 0.29			1.04 ± 0.26		

*Note*: ∆*R* ^2^ (%) represents the change in *R* ^2^ from model 1 to model 2 when >700 m*g* or 700–1500 m*g* activity intensities are added. *B* = unstandardized beta. Model 1 is adjusted for age, sex, height, weight, pubertal status, SEIFA index, muscle cross‐sectional area and PA below 700 m*g* (meaningful activity from 40 m*g* onwards). Model 2 contains all the variables entered into Model 1 with the addition of PA intensity >700 m*g* or 700–1500 m*g*. Model 1 *R* ^2^ = 11.6%, 20.7%, 20.9%, 44.6% and 64.0% for cortical density, trabecular density, endosteal circumference, periosteal circumference and polar SSI, respectively (all *p*'s < 0.001).

Abbreviations: BMD, bone mineral density; PA, physical activity; SEIFA, Socio‐economic Indicators for Areas 2011 Index of Relative Socio‐economic Disadvantage.

^a^ *n* = 770.

^b^ *n* = 762.

Associations with PA became non‐significant for all bone outcomes from >1500 m*g* onwards. The study sample spent 3.28 (±2.83) minutes/day engaged in PA above this intensity. Further regression analyses were performed for each bone outcome and time spent between 700 and 1500 m*g* to determine whether the removal of non‐bone associated PA (e.g., due to clapping or other rapid hand movements) improved the strength of associations observed. Removing activity >1500 m*g* led to a slight increase in mean *R* ^2^ change for activity between 700 and 1500 m*g* (0.92%–1.04%; Table 2). Like >700 m*g*, activity between 700 and 1500 m*g* was associated with better trabecular BMD, bone size and bone strength (Table 2). The mean time spent in activity between 700 and 1500 m*g* was 7.61 (±4.59) minutes per day. Results for the volume analyses (which gives value to short but meaningful amounts of high‐intensity activity) followed the same pattern as the analyses for the time spent in each intensity threshold (data not shown) and did not reveal any additional information regarding associations between bone outcomes and short bursts of high‐intensity activity. The analyses conducted on time spent are presented as they lend themselves to easier interpretation.

## 4 DISCUSSION

Using open‐source software and applying an outcome‐specific approach to the processing and analysis of wrist‐worn raw accelerometry data, this study has demonstrated that PA >700 m*g* was positively and most strongly associated with pQCT‐derived measures of bone density, geometry and strength in a large sample of 11–12‐year‐olds. The use of 1‐s epochs (the shortest epoch currently used in PA research) reduces the risk of misclassifying short, sporadic bursts of high‐impact activity (Aadland et al., 2020) and investigating associations with incremental 50 m*g* intensity thresholds (rather than applying traditional intensity cut‐points a priori) enabled a bone‐specific, beneficial intensity to be identified in this sample. Unlike more traditional approaches, the methods outlined provide a means for more precisely assessing free‐living, bone‐relevant PA and use open‐source software that permits the transparent and reproducible analysis of raw accelerometry data (Migueles et al., 2019), which will enhance the ability to quantify dose‐response associations in the future (Rowlands et al., 2018).

The beneficial intensity of >700 m*g* identified in the present study is equivalent to the VPA threshold identified in calibration studies using wrist‐worn raw accelerometry in children (Hildebrand et al., 2014; Phillips et al., 2013). Several previous cross‐sectional studies have also reported that accelerometer‐derived VPA is most strongly associated with bone outcomes in comparison to other pre‐defined PA intensities (e.g., MPA and MVPA) (Brailey et al., 2022). However, variability in the accelerometry methods used, particularly in the numerous cut‐point definitions employed and averaging of count‐based outputs over long epochs, heavily influences the PA data obtained (Bornstein et al., 2011; Nilsson et al., 2002; Routen et al., 2012), precluding comparisons between studies and preventing more precise amounts and intensities (and types of activity these likely represent) from being identified. Processing the data in smaller 50 m*g* intensity increments instead of pre‐defined intensity thresholds enabled a bone‐relevant intensity to be identified and using reference acceleration ranges to translate findings post‐hoc helped to provide context and an interpretable health message (Rowlands, 2018). In the present sample, the strongest associations with bone outcomes came from activity between 700 and 1500 m*g*, which has been shown to represent running at around 10 km/h in children and adolescents (Hildebrand et al., 2014; Phillips et al., 2013; Rowlands et al., 2019). To our knowledge, there are no other studies that have used raw acceleration averaged over 1‐s epochs to identify a bone‐specific intensity of PA in this population. However, in support of our findings, Deere and colleagues (Deere et al., 2012) assessed the number of impacts per day across a range of impact intensities from raw acceleration and found that impacts >4.2 g, which were also shown to approximate running at ∼10 km/h, were positively associated with hip BMD in older adolescents. Medium‐paced running is a simple, accessible activity that can be easily incorporated into activities of daily living and may therefore be a realistic and achievable option that can be promoted to improve bone health in this population.

In the present study, no significant associations were identified with activity >1500 m*g*. Acceleration values of >1500–2000 m*g* pertain to faster running (∼15 km/h) and >2000 m*g* to sprinting and jumping in children and adolescents (Rowlands et al., 2019). The lack of significant associations from >1500 m*g* is surprising as the osteogenic benefits of high‐impact jumping activities have consistently been demonstrated (Behringer et al., 2014). The mean time spent in activity >1500 m*g* was 3.3 min/day and 80%, 59% and 43% of the sample achieved at least 1, 2 or 3 min/day in this range. Therefore, the lack of association is unlikely due to a lack of statistical power. Instead, it is likely a result of little jumping activity occurring and the wrist‐worn accelerometer capturing non‐bone associated activities involving rapid hand movements but little body movement (e.g., from interactive computer games and clapping) rather than sporadically performed osteogenic activities, such as jumping. This is further supported by the fact that the incremental analysis using volume variables (which give more value to brief periods of high acceleration magnitudes) did not reveal any significant associations with higher acceleration magnitudes. However, a reliance on calibration studies to infer activity type means we cannot be certain about the activity that contributed to the time spent >1500 m*g* and why no significant associations were identified.

Counter to other observations in this study, PA was negatively associated with cortical BMD. This has previously been reported in the same cohort using compositional (Dumuid et al., 2020) and non‐compositional (Osborn et al., 2018) analyses of MVPA and is likely due to the accelerated rate of remodeling that occurs around peak growth (Parfitt, 1994) and in response to mechanical loading from PA (Liu et al., 2003), which causes a transitory reduction in cortical BMD (Parfitt, 1994). When subjected to mechanical loads, bone improves its mechanical competence through geometric adaptations (increases in size) at the expense of density to ensure that the resulting structure is as strong as possible with an economic amount of material (Liu et al., 2003). This supports our findings of greater periosteal circumference and bone strength with increased PA despite reduced cortical density.

The methods outlined in the present study provide a simple, accessible way of assessing free‐living bone‐relevant PA. The open‐source software used (GGIR) can be operated without significant prior programing expertize and can be easily applied to large datasets. Further, using demographic‐specific acceleration ranges to translate findings rather than imposing pre‐defined cut‐points a‐priori helps to maintain comparability of findings and facilitate the development of interpretable health messages (Rowlands, 2018). Nonetheless, these methods are still limited by the fact that they are unable to obtain information regarding activity type and continue to underutilize the richness of the raw signal by aggregating the data into epochs. However, more sophisticated approaches to characterizing PA behavior require complex analytical processes and in the short‐term, are unlikely to take the place of these approaches.

This study is also limited by the cross‐sectional design, which means it may be susceptible to reverse causality. Moreover, whilst several important confounders were controlled for, it was not possible to include information on nutrition, medical conditions or medication, all of which may impact bone. The removal of accelerometers for certain sports may have also impacted the PA data obtained; however, sensitivity analyses removing those reporting more than on average 20, 30 or 60 min/d sport demonstrated that >700 m*g* was still most strongly associated with bone outcomes, and the impact on results was likely minimal. To provide context to the optimal intensity, activity type was inferred from calibration studies, which include a limited range of activities (e.g., sitting, standing, walking and running at different speeds) and may not represent all of the activities being performed in this intensity range. However, sitting, standing and dynamic physical activities, such as walking and running, make up the majority of waking hours in most people and therefore, contribute to a large portion of daily activity (de Almeida Mendes et al., 2018; Hildebrand et al., 2014).

Whilst 1‐s epochs identify a higher proportion of the sporadic, high‐intensity activity performed by children and adolescents (Aadland et al., 2020), activities, such as jumping, occur over a much shorter time frame. Therefore, the averaging of raw data over 1‐s epochs may still underestimate the existence and intensity of these important osteogenic activities. This underestimation may have also been why associations were non‐significant >1500 m*g*. Whilst the use of raw acceleration to monitor impact peaks in relation to bone has been demonstrated at the hip (Haapala et al., 2022), future research should investigate whether it is possible to use this approach with wrist‐worn devices, which are less burdensome and have significantly greater wear compliance (Fairclough et al., 2016). The osteogenic indices obtained from analyzing raw data in this way are also not immediately interpretable, making it difficult to translate study findings into an understandable health message. Therefore, raw acceleration calibration studies are also needed to obtain reference acceleration ranges for impact loading and enable translation of findings from studies using this approach. Since changes in height, body composition and movement patterns that occur during maturation can impact accelerometer output (Welk, 2005), raw acceleration calibration studies should also consider whether maturation impacts the acceleration values obtained for a number of activities, and whether maturity‐specific acceleration values should be applied when translating study findings. Whatever future approaches might be employed to analyze raw data, an ability to either translate raw acceleration peaks via calibration studies into meaningful information regarding activity type or make an adjustment for differences in sample population when using algorithms designed to recognize activity type will likely be necessary.

### 4.1 Conclusions

To conclude, using a more precise, outcome‐specific approach to process and analyze wrist‐worn activity data, we found that ∼10 min/day of activity >700 m*g* (an intensity equivalent to running ∼10 km/h) (Rowlands et al., 2019) is associated with better bone density, geometry and strength in a population‐based sample of 11–12‐year‐olds. The methods outlined can be easily replicated using open‐source software, enabling associations between PA and bone outcomes to be examined and compared in other samples, which has not been possible with commonly used accelerometry methods. Greater comparability of study findings will help to significantly advance understanding of the PA‐bone dose‐response relationship, which in turn, will enable the provision of clear guidance to parents, schools and policy‐makers as to the most time‐economic recommendations for PA to benefit bone health in children at this critical prevention juncture in the course of life.

## CONFLICT OF INTEREST STATEMENT

The authors declare no conflicts of interest.

## Data Availability

The LSAC and CheckPoint data are available under license at https://growingupinaustralia.gov.au/data‐and‐documentation/accessing‐lsac‐data. To access raw accelerometry data, please contact the MCRI's LifeCourse initiative https://lifecourse.melbournechildrens.com.

## References

[ejsc12122-bib-0001] Aadland, Eivind , Lars Bo Andersen , Sigmund Alfred Anderssen , Geir Kåre Resaland , and Olav Martin Kvalheim . 2020. “Accelerometer Epoch Setting Is Decisive for Associations Between Physical Activity and Metabolic Health in Children.” Journal of Sports Sciences 38(3): 256–263. 10.1080/02640414.2019.1693320. 31735120

[ejsc12122-bib-0002] Bailey, D. A. , H. A. McKay , R. L. Mirwald , P. R. E. Crocker , and R. A. Faulkner . 1999. “A Six‐Year Longitudinal Study of the Relationship of Physical Activity to Bone Mineral Accrual in Growing Children: The University of Saskatchewan Bone Mineral Accrual Study.” Journal of Bone and Mineral Research 14(10): 1672–1679. 10.1359/jbmr.1999.14.10.1672. 10491214

[ejsc12122-bib-0003] Bailey, Robert C. , Jodi Olson , Sara L. Pepper , Janos Porszasz , Thomas J. Barstow , and Dan M. Cooper . 1995. “The Level and Tempo of Children's Physical Activities: An Observational Study.” Medicine & Science in Sports & Exercise 27(7): 1033–1041. 10.1249/00005768-199507000-00012. 7564970

[ejsc12122-bib-0004] Baquet, Georges , Gareth Stratton , Emmanuel Van Praagh , and Serge Berthoin . 2007. “Improving Physical Activity Assessment in Prepubertal Children with High‐Frequency Accelerometry Monitoring: A Methodological Issue.” Preventive Medicine 44(2): 143–147. 10.1016/j.ypmed.2006.10.004. 17157370

[ejsc12122-bib-0005] Baxter‐Jones, Adam D. G. , Robert A. Faulkner , Mark R. Forwood , Robert L. Mirwald , and Donald A. Bailey . 2011. “Bone Mineral Accrual From 8 to 30 Years of Age: An Estimation of Peak Bone Mass.” Journal of Bone and Mineral Research 26: 1729–1739. 10.1002/jbmr.412. 21520276

[ejsc12122-bib-0006] Behringer, Michael , Sebastian Gruetzner , Molly McCourt , and Joachim Mester . 2014. “Effects of Weight‐Bearing Activities on Bone Mineral Content and Density in Children and Adolescents: A Meta‐Analysis.” Journal of Bone and Mineral Research 29(2): 467–478. 10.1002/jbmr.2036. 23857721

[ejsc12122-bib-0007] Biggin, Andrew , Julie N. Briody , Kim A. Ramjan , Anna Middleton , M.‐Clare A. Waugh , and Craig F. Munns . 2013. “Evaluation of Bone Mineral Density and Morphology Using pQCT in Children After Spinal Cord Injury.” Developmental Neurorehabilitation 16(6): 391–397. 10.3109/17518423.2012.762590. 23477616

[ejsc12122-bib-0008] Blew, Robert M. , Vinson R. Lee , Joshua N. Farr , Daniel J. Schiferl , and Scott B. Going . 2014. “Standardizing Evaluation of pQCT Image Quality in the Presence of Subject Movement: Qualitative Versus Quantitative Assessment.” Calcified Tissue International 94(2): 202–211. 10.1007/s00223-013-9803-x. 24077875 PMC3949118

[ejsc12122-bib-0009] Bonjour, J.‐Philippe , Thierry Chevalley , Serge Ferrari , and René Rizzoli . 2009. “The Importance and Relevance of Peak Bone Mass in the Prevalence of Osteoporosis.” Salud Publica de Mexico 51: S5–S17. 10.1590/s0036-36342009000700004. 19287894

[ejsc12122-bib-0010] Bornstein, Daniel B. , Michael W. Beets , Wonwoo Byun , Greg Welk , Matteo Bottai , Marsha Dowda , and Russell Pate . 2011. “Equating Accelerometer Estimates of Moderate‐To‐Vigorous Physical Activity: In Search of the Rosetta Stone.” Journal of Science and Medicine in Sport 14(5): 404–410. 10.1016/j.jsams.2011.03.013. 21524938 PMC3158964

[ejsc12122-bib-0011] Brailey, G. , B. Metcalf , R. Lear , L. Price , S. Cumming , and V. Stiles . 2022. “A Comparison of the Associations Between Bone Health and Three Different Intensities of Accelerometer‐Derived Habitual Physical Activity in Children and Adolescents: A Systematic Review (Jan, 10.1007/s00198‐021‐06218‐5, 2022).” Osteoporosis International 33: 1413. 35348838 10.1007/s00198-022-06362-6 PMC9106605

[ejsc12122-bib-0012] Brookes, Denise S. K. , Julie N. Briody , Craig F. Munns , Peter S. W. Davies , and Rebecca J. Hill . 2015. “Cystic Fibrosis‐Related Bone Disease in Children: Examination of Peripheral Quantitative Computed Tomography (pQCT) Data.” Journal of Cystic Fibrosis 14(5): 668–677. 10.1016/j.jcf.2015.04.005. 25957706

[ejsc12122-bib-0013] Brooksgunn, J. , Michelle P. Warren , James Rosso , and Janine Gargiulo . 1987. “Validity of Self‐Report Measures of Girls Pubertal Status.” Child Development 58(3): 829–841. 10.2307/1130220. 3608653

[ejsc12122-bib-0014] Clifford, S. , S. Davies , A. Gillespie , K. Lange , and M. Wake . 2018. Longitudinal Study of Australian Children's Child Health Checkpoint Data User Guide. Melbourne: Murdoch Children's Research Institute.

[ejsc12122-bib-0015] Clifford, Susan A. , Sarah Davies , Melissa Wake and on behalf of the Child Health CheckPoint Team . 2019. “Child Health Checkpoint: Cohort Summary and Methodology of a Physical Health and Biospecimen Module for the Longitudinal Study of Australian Children.” BMJ Open 9(Suppl 3): 3–22. 10.1136/bmjopen-2017-020261. PMC6624028 31273012

[ejsc12122-bib-0016] Cooper, Ashley R. , Anna Goodman , Angie S. Page , Lauren B. Sherar , Dale W. Esliger , Esther M. F. van Sluijs , Lars Bo Andersen , et al. 2015. “Objectively Measured Physical Activity and Sedentary Time in Youth: The International Children’s Accelerometry Database (ICAD).” International Journal of Behavioral Nutrition and Physical Activity 12: 1–10. 10.1186/s12966-015-0274-5. 26377803 PMC4574095

[ejsc12122-bib-0017] da Silva, Inácio C. M. , Vincent T. van Hees , Virgílio V. Ramires , Alan G. Knuth , Renata M. Bielemann , Ulf Ekelund , Soren Brage , and Pedro C. Hallal . 2014. “Physical Activity Levels in Three Brazilian Birth Cohorts as Assessed with Raw Triaxial Wrist Accelerometry.” International Journal of Epidemiology 43(6): 1959–1968. 10.1093/ije/dyu203. 25361583 PMC4276065

[ejsc12122-bib-0018] de Almeida Mendes, Márcio , Inácio C. M. da Silva , Virgílio V. Ramires , Felipe F. Reichert , Rafaela C. Martins , and Elaine Tomasi . 2018. “Calibration of Raw Accelerometer Data to Measure Physical Activity: A Systematic Review.” Gait & Posture 61: 98–110. 10.1016/j.gaitpost.2017.12.028. 29324298

[ejsc12122-bib-0019] Deere, Kevin , Adrian Sayers , Jörn Rittweger , and Jon H. Tobias . 2012. “Habitual Levels of High, But Not Moderate or Low, Impact Activity Are Positively Related to Hip Bmd and Geometry: Results From a Population‐Based Study of Adolescents.” Journal of Bone and Mineral Research 27(9): 1887–1895. 10.1002/jbmr.1631. 22492557 PMC3465797

[ejsc12122-bib-0020] Dumuid, Dorothea , Peter Simm , Melissa Wake , David Burgner , Markus Juonala , Feitong Wu , Costan G. Magnussen , and Timothy Olds . 2020. “The "goldilocks Day" for Children's Skeletal Health: Compositional Data Analysis of 24‐Hour Activity Behaviors.” Journal of Bone and Mineral Research 35(12): 2393–2403. 10.1002/jbmr.4143. 32730680

[ejsc12122-bib-0021] Edwards, B . 2014. “Growing up in Australia: The Longitudinal Study of Australian Children: Entering Adolescence and Becoming a Young Adult.” Family Matters: 5–14.

[ejsc12122-bib-0022] Fairclough, Stuart J. , Robert Noonan , Alex V. Rowlands , Vincent Van Hees , Zoe Knowles , and Lynne M. Boddy . 2016. “Wear Compliance and Activity in Children Wearing Wrist‐ and Hip‐Mounted Accelerometers.” Medicine & Science in Sports & Exercise 48(2): 245–253. 10.1249/mss.0000000000000771. 26375253

[ejsc12122-bib-0023] Farr, Joshua N. , Janet L. Funk , Zhao Chen , Jeffrey R. Lisse , Robert M. Blew , Vinson R. Lee , Monica Laudermilk , Timothy G. Lohman , and Scott B. Going . 2011. “Skeletal Muscle Fat Content Is Inversely Associated with Bone Strength in Young Girls.” Journal of Bone and Mineral Research 26(9): 2217–2225. 10.1002/jbmr.414. 21544865 PMC4414314

[ejsc12122-bib-0024] Fraysse, François , Anneke C. Grobler , Josh Muller , Melissa Wake , and Timothy Olds . 2019. “Physical Activity and Sedentary Activity: Population Epidemiology and Concordance in Australian Children Aged 11–12 Years and Their Parents.” BMJ Open 9(Suppl 3): 136–146. 10.1136/bmjopen-2018-023194. PMC6624037 31273024

[ejsc12122-bib-0025] Freedson, Patty , David Pober , and Kathleen F. Janz . 2005. “Calibration of Accelerometer Output for Children.” Medicine & Science in Sports & Exercise 37(11): S523–S530. 10.1249/01.mss.0000185658.28284.ba. 16294115

[ejsc12122-bib-0026] Gray, M. , and A. Sanson . 2005. “Growing Up in Australia: The Longitudinal Study of Australian Children.” Family Matters: 4–9.

[ejsc12122-bib-0027] Haapala, E. A. , T. Rantalainen , K. D. Hesketh , C. P. Rodda , and R. L. Duckham . 2022. “Accelerometer‐Based Osteogenic Indices, Moderate‐To‐Vigorous and Vigorous Physical Activity, and Bone Traits in Adolescents.” Journal of Musculoskeletal and Neuronal Interactions 22: 514. 36458389 PMC9716299

[ejsc12122-bib-0028] Harris, Paul A. , Robert Taylor , Brenda L. Minor , Veida Elliott , Michelle Fernandez , Lindsay O'Neal , Laura McLeod , et al. 2019. “The Redcap Consortium: Building an International Community of Software Platform Partners.” Journal of Biomedical Informatics 95: 103208. 10.1016/j.jbi.2019.103208. 31078660 PMC7254481

[ejsc12122-bib-0029] Harris, Paul A. , Robert Taylor , Robert Thielke , Jonathon Payne , Nathaniel Gonzalez , and Jose G. Conde . 2009. “Research Electronic Data Capture (Redcap)—A Metadata‐Driven Methodology and Workflow Process for Providing Translational Research Informatics Support.” Journal of Biomedical Informatics 42(2): 377–381. 10.1016/j.jbi.2008.08.010. 18929686 PMC2700030

[ejsc12122-bib-0030] Hildebrand, Maria , Vincent T. Van Hees , Bjorge Hermann Hansen , and Ulf Ekelund . 2014. “Age Group Comparability of Raw Accelerometer Output From Wrist‐ and Hip‐Worn Monitors.” Medicine & Science in Sports & Exercise 46(9): 1816–1824. 10.1249/mss.0000000000000289. 24887173

[ejsc12122-bib-0031] Johnell, O. , and J. A. Kanis . 2006. “An Estimate of the Worldwide Prevalence and Disability Associated with Osteoporotic Fractures.” Osteoporosis International 17(12): 1726–1733. 10.1007/s00198-006-0172-4. 16983459

[ejsc12122-bib-0032] Kalkwarf, H. J. , T. Laor , and J. A. Bean . 2011. “Fracture Risk in Children with a Forearm Injury Is Associated with Volumetric Bone Density and Cortical Area (By Peripheral QCT) and Areal Bone Density (By DXA).” Osteoporosis International 22(2): 607–616. 10.1007/s00198-010-1333-z. 20571770 PMC3298088

[ejsc12122-bib-0033] Liu, LiJing , Ryouko Maruno , Tomoko Mashimo , Kazunori Sanka , Tai Higuchi , Kazuhiko Hayashi , Yoshio Shirasaki , Naoki Mukai , Shinichi Saitoh , and Kumpei Tokuyama . 2003. “Effects of Physical Training on Cortical Bone at Midtibia Assessed by Peripheral QCT.” Journal of Applied Physiology 95(1): 219–224. 10.1152/japplphysiol.01055.2002. 12598486

[ejsc12122-bib-0034] Marfell‐Jones, M. , T. Olds , A. Stewart , and L. Carter . 2006. International Standards for Anthropometric Assessment. Potchefstroom: North‐West University.

[ejsc12122-bib-0035] Migueles, Jairo H. , Alex V. Rowlands , Florian Huber , Séverine Sabia , and Vincent T. van Hees . 2019. “GGIR: A Research Community–Driven Open Source R Package for Generating Physical Activity and Sleep Outcomes from Multi‐Day Raw Accelerometer Data.” Journal for the Measurement of Physical Behaviour 2(3): 188–196. 10.1123/jmpb.2018-0063.

[ejsc12122-bib-0036] Moyer‐Mileur, Laurie J. , Jody L. Quick , and Mary A. Murray . 2008. “Peripheral Quantitative Computed Tomography of the Tibia: Pediatric Reference Values.” Journal of Clinical Densitometry 11(2): 283–294. 10.1016/j.jocd.2007.11.002. 18164637

[ejsc12122-bib-0037] Nilsson, Andreas , Ulf Ekelund , Agneta Yngve , and Michael Söström . 2002. “Assessing Physical Activity Among Children with Accelerometers Using Different Time Sampling Intervals and Placements.” Pediatric Exercise Science 14(1): 87–96. 10.1123/pes.14.1.87.

[ejsc12122-bib-0038] Osborn, William , Peter Simm , Tim Olds , Kate Lycett , Fiona K. Mensah , Josh Muller , Francois Fraysse , et al. 2018. “Bone Health, Activity and Sedentariness at Age 11‐12 Years: Cross‐Sectional Australian Population‐Derived Study.” Bone 112: 153–160. 10.1016/j.bone.2018.04.011. 29674127

[ejsc12122-bib-0039] Parfitt, A. M . 1994. “The Two Faces of Growth: Benefits and Risks to Bone Integrity.” Osteoporosis International 4(6): 382–398. 10.1007/bf01622201. 7696836

[ejsc12122-bib-0040] Petersen, Anne C. , Lisa Crockett , Maryse Richards , and Andrew Boxer . 1988. “A Self‐Report Measure of Pubertal Status‐Reliability, Validity and Initial Norms.” Journal of Youth and Adolescence 17(2): 117–133. 10.1007/bf01537962. 24277579

[ejsc12122-bib-0041] Phillips, Lisa R. S. , Gaynor Parfitt , and Alex V. Rowlands . 2013. “Calibration of the GENEA Accelerometer for Assessment of Physical Activity Intensity in Children.” Journal of Science and Medicine in Sport 16(2): 124–128. 10.1016/j.jsams.2012.05.013. 22770768

[ejsc12122-bib-0042] Routen, Ash C. , Dominic Upton , Martin G. Edwards , and Derek M. Peters . 2012. “Discrepancies in Accelerometer‐Measured Physical Activity in Children Due to Cut‐Point Non‐Equivalence and Placement Site.” Journal of Sports Sciences 30(12): 1303–1310. 10.1080/02640414.2012.709266. 22856351

[ejsc12122-bib-0043] Rowlands, Alex V . 2018. “Moving Forward with Accelerometer‐Assessed Physical Activity: Two Strategies to Ensure Meaningful, Interpretable, and Comparable Measures.” Pediatric Exercise Science 30(4): 450–456. 10.1123/pes.2018-0201. 30304982

[ejsc12122-bib-0044] Rowlands, Alex V. , Stuart J. Fairclough , T. O. M. Yates , Charlotte L. Edwardson , Melanie Davies , Fehmidah Munir , Kamlesh Khunti , and Victoria H. Stiles . 2019. “Activity Intensity, Volume, and Norms: Utility and Interpretation of Accelerometer Metrics.” Medicine & Science in Sports & Exercise 51(11): 2410–2422. 10.1249/mss.0000000000002047. 31318713

[ejsc12122-bib-0045] Rowlands, Alex V. , Evgeny M. Mirkes , Tom Yates , Stacey Clemes , Melanie Davies , Kamlesh Khunti , and Charlotte L. Edwardson . 2018. “Accelerometer‐assessed Physical Activity in Epidemiology: Are Monitors Equivalent?” Medicine & Science in Sports & Exercise 50(2): 257–265. 10.1249/mss.0000000000001435. 28976493

[ejsc12122-bib-0046] Rowlands, Alex V. , Tom Yates , Melanie Davies , Kamlesh Khunti , and Charlotte L. Edwardson . 2016. “Raw Accelerometer Data Analysis with GGIR R‐Package: Does Accelerometer Brand Matter?” Medicine & Science in Sports & Exercise 48(10): 1935–1941. 10.1249/mss.0000000000000978. 27183118

[ejsc12122-bib-0047] Sherk, Vanessa D. , Debra A. Bemben , Michael G. Bemben , and Mark A. Anderson . 2012. “Age and Sex Differences in Tibia Morphology in Healthy Adult Caucasians.” Bone 50(6): 1324–1331. 10.1016/j.bone.2012.03.005. 22449446 PMC4082662

[ejsc12122-bib-0048] Statistics ABo . 2011. Census of Population and Housing: Socio‐Economic Indexes for Areas (SEIFA). Australian Bureau of Statistics. https://www.abs.gov.au/websitedbs/censushome.nsf/home/seifa2011

[ejsc12122-bib-0049] Stiles, Victoria H. , Brad S. Metcalf , Karen M. Knapp , and Alex V. Rowlands . 2017. “A Small Amount of Precisely Measured High‐Intensity Habitual Physical Activity Predicts Bone Health in Pre‐ and Post‐Menopausal Women in UK Biobank.” International Journal of Epidemiology 46(6): 1847–1856. 10.1093/ije/dyx080. 29106579 PMC5837700

[ejsc12122-bib-0050] Stiles, Victoria H. , Matthew Pearce , Isabel S. Moore , Joss Langford , and Alex V. Rowlands . 2018. “Wrist‐worn Accelerometry for Runners: Objective Quantification of Training Load.” Medicine & Science in Sports & Exercise 50(11): 2277–2284. 10.1249/mss.0000000000001704. 30067593 PMC6195805

[ejsc12122-bib-0051] Trost, Stewart G . 2020. “Population‐level Physical Activity Surveillance in Young People: Are Accelerometer‐Based Measures Ready for Prime Time?” International Journal of Behavioral Nutrition and Physical Activity 17(1): 28. 10.1186/s12966-020-00929-4. 32183807 PMC7079381

[ejsc12122-bib-0052] Turner, Charles H. , and Alexander G. Robling . 2003. “Designing Exercise Regimens to Increase Bone Strength.” Exercise and Sport Sciences Reviews 31(1): 45–50. 10.1097/00003677-200301000-00009. 12562170

[ejsc12122-bib-0053] van Hees, Vincent T. , Zhou Fang , Joss Langford , Felix Assah , Anwar Mohammad , Inacio C. M. da Silva , Michael I. Trenell , Tom White , Nicholas J. Wareham , and Søren Brage . 2014. “Autocalibration of Accelerometer Data for Free‐Living Physical Activity Assessment Using Local Gravity and Temperature: An Evaluation on Four Continents.” Journal of Applied Physiology 117(7): 738–744. 10.1152/japplphysiol.00421.2014. 25103964 PMC4187052

[ejsc12122-bib-0054] van Hees, Vincent T. , Lukas Gorzelniak , Emmanuel Carlos Dean León , Martin Eder , Marcelo Pias , Salman Taherian , Ulf Ekelund , et al. 2013. “Separating Movement and Gravity Components in an Acceleration Signal and Implications for the Assessment of Human Daily Physical Activity.” PLoS One 8(4): e61691. 10.1371/journal.pone.0061691. 23626718 PMC3634007

[ejsc12122-bib-0055] Vlok, Jennifer , Peter J. Simm , Kate Lycett , Susan A. Clifford , Anneke C. Grobler , Katherine Lange , Najmi Ismail , William Osborn , and Melissa Wake . 2019. “Pqct Bone Geometry and Strength: Population Epidemiology and Concordance in Australian Children Aged 11–12 Years and Their Parents.” BMJ Open 9(Suppl 3): 63–74. 10.1136/bmjopen-2018-022400. PMC6624036 31273017

[ejsc12122-bib-0056] Weaver, C. M. , C. M. Gordon , K. F. Janz , H. J. Kalkwarf , J. M. Lappe , R. Lewis , M. O’Karma , T. C. Wallace , and B. S. Zemel . 2016. “The National Osteoporosis Foundation's Position Statement on Peak Bone Mass Development and Lifestyle Factors: A Systematic Review and Implementation Recommendations.” Osteoporosis International 27(4): 1281–1386. 10.1007/s00198-015-3440-3. 26856587 PMC4791473

[ejsc12122-bib-0057] Welk, Gregory J . 2005. “Principles of Design and Analyses for the Calibration of Accelerometry‐Based Activity Monitors.” Medicine & Science in Sports & Exercise 37(11): S501–S511. 10.1249/01.mss.0000185660.38335.de. 16294113

